# Body Composition Impact on Sleep in Young Adults: The Mediating Role of Sedentariness, Physical Activity, and Diet

**DOI:** 10.3390/jcm9051560

**Published:** 2020-05-21

**Authors:** Almudena Carneiro-Barrera, Francisco J. Amaro-Gahete, Francisco M. Acosta, Jonatan R. Ruiz

**Affiliations:** 1 Sleep and Health Promotion Laboratory, Mind, Brain and Behaviour Research Centre (CIMCYC), University of Granada, 18011 Granada, Spain; 2 EFFECTS-262 Research Group, Department of Medical Physiology, School of Medicine, University of Granada, 18071 Granada, Spain; amarof@ugr.es; 3 Department of Health, Camilo José Cela University, 28692 Madrid, Spain; 4 PROmoting FITness and Health through Physical Activity Research Group (PROFITH), Sport and Health University Research Institute (iMUDS), Department of Physical Education and Sports, Faculty of Sport Sciences, University of Granada, 18071 Granada, Spain; acostaf@ugr.es (F.M.A.); ruizj@ugr.es (J.R.R.)

**Keywords:** body composition, sleep, obesity, sedentariness, physical activity, diet

## Abstract

Obesity and sleep disturbances are both related to endocrine and metabolic alterations, cardiovascular disease, and impaired daytime functioning and mood. However, the bidirectional relationship between these conditions and the underlying mechanisms still remain unclear. This study aimed to investigate the potential association of anthropometric and body composition parameters with sleep in young adults, considering the mediating role of sedentariness, physical activity, and diet. A total of 187 adults aged 18–25 (35.29% men) participated in the study. Body mass index (BMI), waist–hip ratio, and waist–height ratio were calculated, and a dual-energy X-ray absorptiometry scanner was used to assess body composition. Sedentary time and physical activity, as well as sleep duration and quality, were objectively and subjectively measured using accelerometry and the Pittsburgh Sleep Quality Index. An inverse association was found between BMI and total sleep time (*β* = −0.165, *p* = 0.029). Waist–hip ratio and lean mass index were also negatively associated with total sleep time (*β* = −0.222, *p* = 0.007, and *β* = −0.219, *p* = 0.004) and sleep efficiency (*β* = −0.174, *p* = 0.037, and *β* = −0.188, *p* = 0.013). Sedentary time moderated by sex explained the association of BMI with total sleep time such that a high BMI was related to higher sedentariness in men which, in turn, was significantly associated with shorter sleep duration. Sedentary time is, therefore, a link/risk factor mediating the association of high BMI with short sleep duration in healthy young men.

## 1. Introduction

The obesity epidemic is globally recognized as a substantial health concern in modern society, becoming an important risk factor for increased morbidity and mortality with vast clinical and economic adverse consequences on the health system [1]. According to the Global Burden of Diseases, Injuries, and Risk Factors Study 2017 [2], the global exposure risk to a high body mass index (BMI) ominously increased by 70.39% since 1990 in the overall population. In 2017, 4.72 million deaths (36.3% higher since 2007) and 148 million disability-adjusted life-years were attributable to a BMI higher than or equal to 25 kg/m^2^. Therefore, obesity is now the fourth leading risk factor worldwide for mortality, predominantly due to the well-established association between high BMI and cardiovascular diseases [2].

Similarly, sleep duration and quality disturbances are being considered emerging public health issues due to their adverse health-related consequences, increasing rate of prevalence and, thus, their major economic cost [3,4]. Short sleep duration (commonly <7 h per night) [5]—included in the most recent International Classification of Sleep Disorders (ICSD-3) as insufficient sleep syndrome [6]—is also significantly related to a greater risk of mortality from cardiovascular diseases and all causes [7]. Specifically, epidemiologic evidence suggests that short sleepers have up to 12% greater risk of all-cause mortality [8] potentially due to the vast number of severe comorbidities such as obesity and other non-communicable diseases [9,10].

A bidirectional relationship between sleep disorders and obesity is, therefore, widely accepted throughout the body of literature in this field. However, although the effects of short sleep on obesity were shown in clinical and epidemiological research [9,10], the underlying mechanisms explaining the effects of obesity on sleep still remain unclear [11,12]. Potential mediating factors may be sedentariness and physical activity, which are closely related to both obesity and sleep duration and quality [13,14,15]. Those who have a high BMI are usually less active and display greater sitting time and screen viewing, which is inversely associated with sleep quality and duration [15,16]. Regarding physical activity, although it is related to better BMI and sleep duration and quality [17,18], some studies did not find this association in younger ages [19]. These behavioral factors (i.e., sedentariness and physical activity) may, therefore, be significant key/link elements explaining the closed association between BMI and sleep, although there is a need for further research, especially in young adults.

Similarly, dietary patterns were also shown to be related to sleep duration and quality [20,21,22]. According to the evidence, high-carbohydrate intake is related to longer sleep duration and better sleep architecture, whereas high-fat intake and low-protein intake may produce shorter sleep duration, lower sleep efficiency, and increased sleep fragmentation [20,21,22]. As sleep disturbances, in turn, yield higher energy intake and weight gain [9,10], diet quality may also be a mediating factor between body composition and total sleep time.

Apart from BMI, cross-sectional studies also exposed that specific body composition parameters can be closely related to sleep duration and quality. Concretely, lean mass is positively related to sleep duration and quality, whereas a high fat mass seems to be linked to short and poorer sleep [23,24,25]. Indeed, a combination of both low skeletal muscle mass and increased fat mass, i.e., sarcopenic obesity [26], is strongly associated with chronic conditions [27] and, in particular, with short sleep duration [28]. Thus, sarcopenic obesity may not only be a potential risk factor of sleep disorders, but also a syndrome that exacerbates the adverse consequences of these conditions [29].

Understanding the relationship of body weight and composition with sleep duration and quality is, therefore, of clinical interest in order to design appropriate strategies of health promotion in young populations and, in turn, to prevent early morbidity and mortality. Although there are studies showing independent relationships of markers of obesity with sleep and behavioral factors [13,14,15,16], at least one of these outcomes was solely assessed by subjective measures such as questionnaires. Moreover, to the best of our knowledge, there are no studies clarifying the potential mechanisms which may enclose all these key elements in an individual model. Therefore, our study was mainly aimed at elucidating the association of anthropometry and body composition—including BMI, waist–hip ratio (WHiR), waist–height ratio (WHtR), fat mass index, visceral adipose tissue, and lean mass index parameters—with objective and subjective sleep duration and quality in young sedentary adults. Additionally, we also pursued to investigate the potential mediating role of objectively measured sedentariness, physical activity, and diet in the specific relationship between BMI and sleep duration and quality. Particularly, we hypothesized that there would be a significant relationship of anthropometric and body composition parameters with sleep duration and quality and that, in the specific association of BMI and sleep, this relationship would be mediated by sedentariness, physical activity, and/or diet.

## 2. Experimental Section

### 2.1. Study Protocol and Participants

A total of 187 healthy young adults (*n* = 121 women), aged 18–25 years and with a BMI ranging from 18 to 35 kg/m^2^, were selected from the Activating Brown Adipose Tissue through Exercise (ACTIBATE) study (Table 1), an exercise-based randomized controlled trial (ClinicalTrials.gov identifier NCT02365129). A comprehensive explanation of the study design and methodology can be found elsewhere [30]. The inclusion/exclusion criteria were as follows: (i) to be non-smokers, (ii) not to be enrolled in a weight loss program or to be engaged in regular physical activity >20 min on >3 days/week during the prior 12 weeks, (iii) to have a stable body weight (body weight changes <3 kg) over the previous three months, (iv) not to be physically active (<20 min on <3 days/week), (v) not to take any medication or drugs, and (vi) not to suffer from any acute or chronic illness. The study was conducted in the south of Spain between October 2015 and November 2016 at the Sport and Health University Research Institute (iMUDS). The study protocol and written informed consent procedures were accordingly performed with the latest revised Declaration of Helsinki (2013), and they were approved by the Human Research Ethics Committee of the University of Granada (i.e., 924) and Junta de Andalucía.

### 2.2. Outcome Measurements

#### 2.2.1. Sleep Duration and Quality

Objective sleep outcomes were assessed using a wrist-worn accelerometer (ActiGraph GT3X+, Pensacola, FL, USA) continuously 24 h a day for seven consecutive days [23,30,31]. Subjects were instructed to wear the accelerometers on the non-dominant wrist as much as possible, removing them only when swimming or bathing. A seven-day sleep diary was provided to the participants in order to register their bedtime, wake up time, and the time they removed the device every day. The accelerometer was programmed to store raw accelerations at 100 Hz of sampling frequency [32], and its derived raw data were stored and downloaded using the ActiLife software (version 6.13.3, ActiGraph, Pensacola, FL, US). A conversion of the resulted GT3X+ files was performed to get 1” epoch csv files containing *x*, *y*, and *z* vectors to improve raw data processing. Subsequently, these files were processed in R (version 3.1.2, https://www.R-project.org/ [33]) using the GGIR package (version 1.5-12, https://cran.r-project.org/web/packages/GGIR/ [34]) which included (i) a signal auto-calibration using local gravity as a reference [35], (ii) an evaluation of sustained abnormally high values, (iii) a detection of non-wear time, (iv) a calculation of the Euclidean Norm Minus One *g* (ENMO), and (iv) an assessment of waking and sleeping time using an automatized algorithm [36].

Actigraphy recordings were used to determine (i) total sleep time (defined as the total amount of time spent in bed excluding sleep latency), (ii) sleep efficiency (defined as the percentage of sleep time over the bedtime), and (iii) wake after sleep onset (WASO; defined as the sum of time awaken from sleep onset to the final awakening) [37]. Those participants registering less than 16 h/day of wear time for less than four days and/or not having data from at least one weekend day were excluded from the final analysis.

The Spanish-validated version of the Pittsburgh sleep quality index (PSQI) scale was used to determine participant’s subjective sleep quality [38,39]. This scale includes a total of 19 self-rated items combined to form seven component scores (i.e., subjective sleep quality, sleep latency, sleep duration, habitual sleep efficiency, sleep disturbances, use of sleeping medications, and daytime dysfunction), each of which has a range of 0–3 points. The sum of these seven PSQI component scores is considered to obtain a global PSQI score (ranging from 0–21), with higher scores indicating poorer sleep quality.

#### 2.2.2. Anthropometry and Body Composition

Body weight and height were determined with participants wearing light clothing and without shoes using a SECA scale and stadiometer (model 799; Electronic Column Scale, Seca GmbH, Hamburg, Germany). Subsequently, the BMI was calculated as weight (kilograms) divided by height squared (square meters). Waist circumference (centimeters) was assessed midway between the lowest rib and the top of the iliac crest after exhalation, whereas hip circumference (centimeters) was determined over the great trochanters. Each measurement was performed twice and averaged. WHiR and WHtR were subsequently calculated as waist divided by hip circumferences and height, respectively.

Body composition was measured by dual-energy X-ray absorptiometry (Discovery Wi; Hologic, Inc., Marlborough, MA, USA) strictly following the manufacturer’s instructions, obtaining lean mass, fat mass, and visceral adipose tissue mass [40]. Lean mass index and fat mass index were calculated as lean mass (kilograms) and fat mass (kilograms), respectively, divided by height squared (square meters).

#### 2.2.3. Sedentary Time and Physical Activity Intensity Levels

Sedentariness and physical activity levels were determined by accelerometry (see specific details about the procedure above) [41,42]. The GGIR package (v.1.5-12, https://cran.r-project.org/web/packages/GGIR/) in R (v.3.1.2, https://www.cran.r-project.org/) was used to discern among (i) sedentary time, (ii) light physical activity (LPA), (iii) moderate physical activity (MPA), (iv) vigorous physical activity (VPA), and (v) moderate–vigorous physical activity (MVPA), using age-specific thresholds for ENMO [43,44].

#### 2.2.4. Dietary Intake

An average of three 24-h recalls performed on non-consecutive days (including one weekend day) was used to register dietary intake. This technique is considered a valid method to assess energy intake with an 8% to 10% margin of error [45]. The 24-h recalls were conducted by experienced dietitians/nutritionists who obtained a detailed description of the food consumed by the participants through a personal interview. Colored photographs containing different foods and portion sizes were used in order to assist in estimating the quantity of food consumed. Energy intake and macronutrient content (i.e., fat, protein, and carbohydrate intake) were obtained using the EvalFINUT^®^ software (FINUT, Granada, Spain).

### 2.3. Statistical Analysis

Shapiro–Wilk test, quantile–quantile (Q–Q) plots, and visual checking of histograms were used to confirm the normal distribution of all variables. Descriptive characteristics of participants are presented as mean ± standard deviation. Sex differences were determined by unpaired Student’s *t*- tests and Mann–Whitney U tests for non-parametric variables. Given that non-significant sex interaction (*p* < 0.05) was observed in regression analyses, data from men and women were simultaneously included in all the analyses performed.

Pearson and Spearman correlations were preliminarily conducted to examine the relationship among objective and subjective sleep parameters, anthropometric and body composition outcomes, sedentary time, physical activity levels, and dietary intake variables. The associations of anthropometric (i.e., WHiR, WHtR, and BMI) and body composition parameters (i.e., lean mass index, fat mass index, and visceral adipose tissue) with sleep parameters were studied by simple linear regression (Model 0) and multiple linear regression models adjusted by sex (Model 1). The interactions of sedentary time and physical activity in these associations were also studied by linear models.

In order to analyze the potential mediating role of sedentary time, physical activity levels, and dietary intake outcomes in the relationship between BMI and sleep parameters, mediation analyses were performed following the steps for establishing mediation proposed by Baron and Kenny [46], using corresponding models in the most recent PROCESS macro version 3.4 developed by Andrew F. Hayes [47] (http://processmacro.org/version-history.html). According to Baron and Kenny, the independent variable *X* must firstly be associated to the dependent variable *Y* (total effect; path c) and secondly with the mediator variable *M* (path a); thirdly, the mediator variable *M* must also be significantly correlated to the dependent variable *Y* even after adjusting by the causal variable *X* (path b);, fourthly, the effects of *X* on *Y* should drop in strength and/or significance after controlling by *M* (direct effect path c’) for the mediation to be established. Preacher and Hayes’s SPSS macro incorporates the stepwise procedure described by Baron and Kenny and includes the estimation of the indirect effect ab (i.e., ab = c − c’; the amount of mediation or reduction of the effects of *X* on *Y*), which is the recommended way of robustly measuring the mediation [47,48,49]. This latter estimation is based on the bootstrapping method, a non-parametric resampling method which estimates the indirect effect through 5000 bias-corrected bootstrap samples and 95% confidence intervals. If these confidence intervals do not include zero, the indirect effect ab can be considered as different from this value and, therefore, the mediation is assumed.

Calculations were performed using the Statistical Package for the Social Sciences v.22.0, (IBM SPSS Statistics, IBM Corporation, Armonk, NY, USA). GraphPad Prism 5 software (GraphPad Software, San Diego, CA, USA) was used to draw plots. Significance was set at *p* < 0.05.

## 3. Results

Descriptive characteristics of the study participants are shown in Table 1. Overall, men presented statistically significant higher values of WASO, BMI, WHiR, WHtR, lean mass, lean mass index, visceral adipose tissue mass, energy intake, fat intake, protein intake, and carbohydrate intake compared with those observed in women (all *p* < 0.05). In contrast, women showed greater levels of sleep efficiency and fat mass percentage than men. Based on BMI, 60.1% of participants were of normal weight, 24.7% were overweight (BMI 25.0–29.9 kg/m^2^), and 15.2% were classified as obese (BMI ≥30 kg/m^2^).

**Figure 1:**
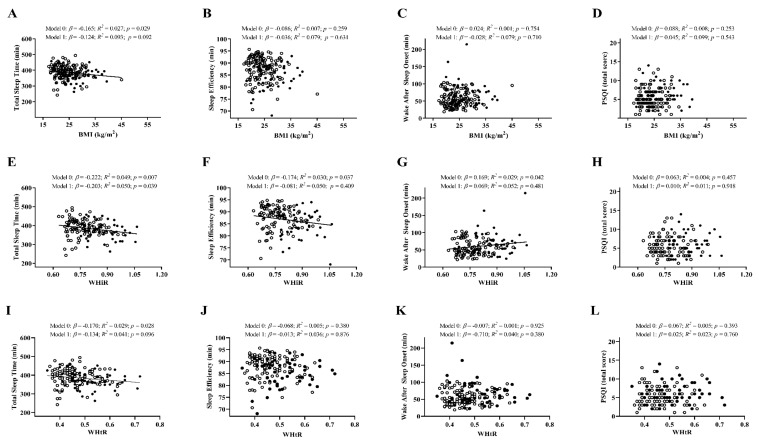
Linear regression graphs of body mass index (BMI), waist–hip ratio (WHiR), and waist–height ratio (WHtR) with total sleep time (Panels (**A**,**E**,**I**)), sleep efficiency (Panels (**B**,**F**,**J**)), wake after sleep onset (Panels (**C**,**G**,**K**)), and PSQI total score (Panels **D**,**H**,**L**)), before and after adjusting by sex (model 0 and model 1, respectively). *β*, standardized linear regression coefficient; *R^2^*, coefficient of determination; *p*, *p*-value. Closed and open circles represent men and women, respectively. Abbreviations: BMI, body mass index; WHiR, waist–hip ratio; WHtR, waist–height ratio; PSQI, Pittsburgh sleep quality index.

**Figure 2:**
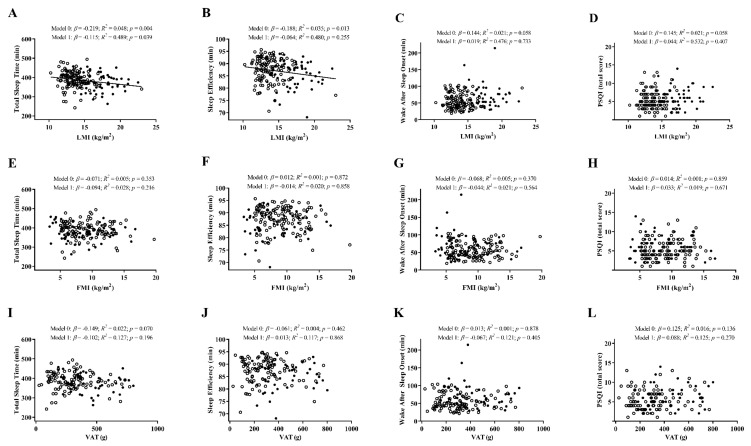
Linear regression graphs of lean mass index (LMI), fat mass index (FMI), and visceral adipose tissue (VAT) with total sleep time (Panels (**A**,**E**,**I**)), sleep efficiency (Panels (**B**,**F**,**J**)), wake after sleep onset (Panels (**C**,**G**,**K**)), and PSQI total score (Panels (**D**,**H**,**L**)), before and after adjusting by sex (model 0 and model 1, respectively). *β*, standardized linear regression coefficient; *R^2^*, coefficient of determination; *p*, *p*-value. Closed and open circles represent men and women, respectively. Abbreviations: LMI, lean mass index; FMI, fat mass index; VAT, visceral adipose tissue mass; PSQI, Pittsburgh sleep quality index.

The associations of anthropometric and body composition outcomes with sleep parameters are shown in Figure 1 and Figure 2, as well as Table S1 (Supplementary Materials). There was a significant negative association between BMI and total sleep time (*β* = −0.165; *R^2^* = 0.027; *p* = 0.029; Figure 1A), which was attenuated after controlling by sex (*p* = 0.092). WHiR was also negatively associated with total sleep time (*β* = −0.222; *R^2^* = 0.049; *p* = 0.007; Figure 1E) and sleep efficiency (*β* = −0.174; *R^2^* = 0.030; *p* = 0.037; Figure 1F) and positively related to WASO (*β* = 0.169; *R^2^* = 0.029; *p* = 0.042; Figure 1G), although these associations disappeared after adjusting by sex in the case of sleep efficiency (*p* = 0.409) and WASO (*p* = 0.481). Similarly, WHtR and total sleep time were negatively associated (*β* = −0.170; *R^2^* = 0.029; *p* = 0.028; Figure 1I), with this association disappearing after adjusting by sex (*p* = 0.096).

Regarding lean mass index, a significant negative association was observed between this outcome and total sleep time (*β* = −0.219; *R^2^* = 0.048; *p* = 0.004; Figure 2A), which persisted after controlling by sex (*p* = 0.039). Similarly, a significant negative association was noted between lean mass index and sleep efficiency (*β* = −0.188; *R^2^* = 0.035; *p* = 0.013; Figure 2B), which disappeared after adjusting by sex (*p* = 0.255). No further associations were found between the remaining anthropometric and body composition parameters with sleep-related outcomes (Figure 1 and Figure 2). When we examined the interaction effects of body composition parameters and sedentary time and/or MVPA on sleep parameters, only lean mass index and sedentary time showed a significant interaction effect with total sleep time (*p* = 0.033).

Correlation analyses for anthropometric and body composition outcomes, sleep parameters, sedentary time, physical activity levels, and dietary intake can be found in Tables S2–S4 (Supplementary Materials). Considering required correlations in order to proceed with the mediational analyses, only sedentary time was found to be related to both BMI and total sleep time. Thus, while a significant positive relationship of BMI and sedentary time was observed only in men (*p* < 0.05; Table S3, Supplementary Materials), a negative correlation was found between sedentary time and total sleep time in both men and women (*p* < 0.01; Table S2, Supplementary Materials). Other relevant correlations found among sleep parameters and dietary and physical activity outcomes were a statistically significant positive relationship between MVPA and sleep efficiency (*p* < 0.05) and an inverse correlation between MVPA and WASO (*p* < 0.05; Table S2, Supplementary Materials).

**Figure 3:**
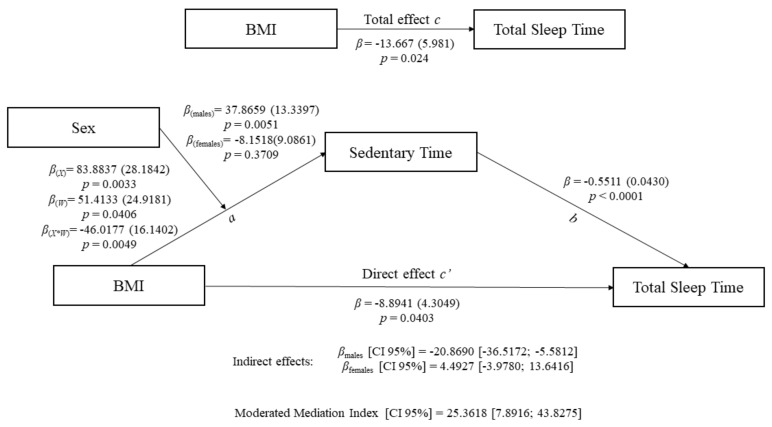
Moderated mediation model of the relationship between body mass index and total sleep time (min) with sedentary time (min) and sex as mediator and moderator variables, respectively. Paths a, b, c, and c’ are presented as unstandardized coefficients (standard error). [Lower-limit confidence interval (CI); upper-limit CI], lower and upper levels for 95% bias-corrected CIs of the indirect effects based on 5000 bootstraps. BMI, body mass index; CI, confidence interval; *β*(X), unstandardized coefficient of the association of BMI with sedentariness; *β*(W), unstandardized coefficient of the association of sex with sedentariness; *β*(X*W), unstandardized coefficient of the association between BMI and sedentariness moderated by sex.

Regarding the mediational analyses, Figure 3 and Table 2 show the moderated mediating effect of sedentary time in the relationship between BMI and total sleep time. As BMI was only significantly and positively related to sedentary time in men, we proceeded to perform a moderated mediation analysis (model 7), including sex as a moderator variable of the mediational effect of sedentary time. Considering this model, BMI was negatively associated with total sleep time (total effect c = −13.667; *p* < 0.05) and positively related to sedentary time only in men (effect a = 37.866; *p* < 0.01). Sedentary time, in turn, was also negatively associated to total sleep time (effect b = −0.551; *p* < 0.01). After including the moderator and mediator variable (i.e., sex and sedentary time, respectively) in the model, the association between BMI and total sleep time, although remaining significant, was significantly reduced (direct effect c’ = −8.894; *p* < 0.05), thus suggesting that a partial moderated mediation was reached. The confidence interval of the conditional indirect effect ab (amount of mediation) for men, as well as the final moderated mediation index (the quantification of the association between the indirect effect and the moderator), did not include the value zero; therefore, the mediating role of sedentary time moderated by sex in the significant relationship between BMI and total sleep time was further confirmed.

The mediating role of physical activity levels and dietary intake in the relationship between BMI and total sleep time was not statistically significant in any case (see Figure S1, Supplementary Materials).

## 4. Discussion

Our study sought to elucidate the potential association of anthropometry and body composition parameters with sleep duration and quality and, in turn, whether behavioral factors such as sedentariness, physical activity, and diet were potential mediating mechanisms explaining the closed association between BMI and sleep in young adults. Out of all included body composition and sleep parameters, only BMI, WHiR, WHtR, and LMI were associated with objective sleep parameters, with these associations being attenuated after adjusting by sex. As we expected, although physical activity and diet did not mediate the relationship between BMI and sleep duration, sedentariness significantly explained the association between these two variables. According to our results, sedentary time moderated by sex partially explained the association of BMI with total sleep time such that a higher BMI was related to higher sedentariness in men which, in turn, was significantly associated with shorter sleep duration.

High BMI and short sleep duration caused by sleep disorders are both public health concerns due to their high and increasing prevalence in the overall population [1,2,3,4] and their broad-ranging adverse health-related consequences such as endocrine and metabolic alterations, life-threating cardiovascular diseases, and impaired daytime functioning and mood [50,51]. Although the bidirectional relationship between obesity and sleep disturbances is well accepted [52], and the effects of short sleep on BMI are widely shown and explained [10,53], the underlying mechanism explaining the effects of high BMI on sleep still remains uncertain [11,12]. According to our results, sedentary time may be one of the mediating factors explaining the negative association of BMI with sleep duration in young men, which is in accordance with previous studies where sedentariness was independently and closely associated with higher BMI and shorter sleep duration [13,14,15,16]. Regarding sex differences, previous epidemiological/experimental studies also showed that the beneficial effects of acute or regular exercise on sleep were significantly higher in men than women [17,54] and, in general, the association between BMI and sleep was lower in women possibly due to other cofounders such as psychological stress rather than exercise/sedentary time [55,56].

Concerning moderate–vigorous physical activity, we did not find a significant association between this variable and total sleep time, which is in accordance with results from a recently published cross-sectional study where an increased moderate–vigorous physical activity, although associated with improved subjective sleep quality, was not related to better or longer objective sleep duration [57]. Thus, differing from sedentary time, physical activity did not explain the association between BMI and objective or subjective sleep, which is consistent with the overwhelming and major negative impact of sedentary behavior on sleep and overall metabolic risk independently of physical activity [15]. Nevertheless, we found that increased moderate–vigorous physical exercise was significantly associated with higher sleep efficiency and lower wake after sleep onset. Therefore, while the practice of physical activity at this intensity may not be related to longer sleep duration, it is related to a reduced number of awakenings or time spent awake after sleep onset which, in turn, is reflected in higher sleep efficiency. These results are also supported by previous empirical evidence which emphasized that, although total sleep time may not be modified by an increased moderate to vigorous physical activity, a reduction in this intensity seems to be linked to reduced slow-wave sleep and, in turn, increased rapid eye movement (REM) sleep [58].

Similarly, dietary intake was previously found to be linked to sleep architecture and quality [20,21,22]. Whereas high-fat intake and low-protein intake seem to produce reductions in REM sleep, increased arousal index and, thus, poorer sleep quality [21,22], high-carbohydrate intake is related to increased REM sleep, as well as improved sleep architecture and quality [20]. However, we did not find any significant relationship between dietary intake—including energy, fat, protein, and carbohydrate intake—and objective or subjective sleep duration and quality in our sample. These results may be explained by the use of accelerometry as a sleep measurement which, although providing reliable measures of objective sleep, does not allow discerning more potential specific changes in sleep architecture rather than duration. The use of dietary recalls for the energy intake estimations may also potentially suffer from significant bias [59,60], which could similarly explain the lack of association between these dietary outcomes and sleep.

Apart from BMI, only WHiR, WHtR, and LMI were related to objectively measured sleep parameters. According to our results, higher values of WHiR and WHtR were associated with shorter sleep duration and, only in the case of WHiR, with lower sleep efficiency and increased time awake after sleep onset. These results are in accordance with previous research emphasizing that, in addition to being major risk factors for cardiometabolic alterations, cancer and all-cause mortality [61,62], WHiR/WHtR and abdominal obesity are significantly related to severe sleep disturbances such as short sleep duration and obstructive sleep apnea [23,63], especially in younger adults [61]. Regarding LMI, we found that those participants with higher LMI exhibited shorter sleep and lower sleep efficiency. Although in contrast to previous studies where LMI was positively associated with sleep quality [23], these results may be explained by evidence exposing that lower LMI was related to longer or excessive sleep duration, potentially due to increased levels of cortisol triggered by sleep disturbances which, in turn, are related to muscle degradation [24]. Therefore, an inverted u-shaped relationship between LMI and sleep duration may occur, although the testing of this association in our sample was not feasible due to the sole inclusion of healthy young adults, i.e., non-inclusion of extreme LMI values.

Interestingly, no body composition parameters were related to subjective sleep quality. Although these results may seem contrary to those regarding BMI and objective sleep duration, they emphasize the well-established discrepancy between objective and subjective sleep [64]. Unlike objective sleep duration, which is the actual time the individual is asleep, subjective sleep quality refers to the self-evaluation or one’s perception of sleep duration and satisfaction, independently of the more objective aspect of sleep. According to the evidence, subjective sleep quality is highly related to the individual’s emotional status and cognitive function [65,66,67], which could explain the non-significant associations found between body composition parameters and subjective sleep, potentially due to underlying uncontrolled psychological cofounders.

To the best of our knowledge, this is the first study acutely analyzing modifiable behavioral risk factors as potential underlying mechanisms through which BMI and sleep may be related, including not only reliable objective measures of body composition and sleep but also the gold-standard test of physical activity, i.e., accelerometry. Apart from independent associations of body composition parameters and physical activity with sleep, we essentially found that sedentary behavior seems to significantly explain part of the adverse impact of high BMI on sleep duration in young men. Our study, therefore, has significant contributions to the clinical and research practice in this field, further clarifying the recognized bidirectional relationship between obesity and sleep disorders [9] and, thus, supporting the need of designing appropriate strategies of health promotion through physical activity and/or sedentary behavior avoidance in young populations. As sedentariness is also independently linked to cardiovascular disease, cancer incidence and mortality, and all-cause mortality in adults [68], modifying this behavioral risk factor could not only improve the current obesity and sleep disorder epidemics in young adults, but also prevent this population from suffering chronic health conditions and early mortality.

## 5. Limitations and Future Research

However, our results should be cautiously interpreted as these are restricted to the sample included, methodology followed, and study design. Although the analysis of mediator variables enables approach to causal inferences [46,47,48,49], our main limitation was the cross-sectional study design which does not allow completely determining cause–effect relationships. The use of accelerometry/actigraphy for the objective measurement of sleep, although widely used and validated [36,37], may have underestimated/overestimated sleep outcomes such as sleep duration, sleep efficiency, sleep onset latency, and wake after sleep onset [32]. Similarly, although dual-energy X-ray absorptiometry is widely validated and used for the measurement of body composition, the use of magnetic resonance imaging—the gold-standard technique—could have provided our study with more accurate body composition data. The use of dietary recalls for the energy intake estimations, although considered a valid method to assess energy intake with an 8–10% margin of error [45], may also potentially suffer from significant bias. Finally, our sample only included healthy young adults; thus, although there is a lack of studies including this population in this field of research, the generalization of our findings may be limited to this population. Future well-designed longitudinal studies should be performed in order to robustly establish causal relationships between body composition parameters and sleep. Furthermore, studies in this field of research should include polysomnography, which is the gold-standard method to appropriately assess not only sleep duration and efficiency but also potential associations between behavioral factors and polysomnographic sleep outcomes such as sleep architecture. Finally, as psychological conditions such as mood and anxiety disturbances are related to both obesity and sleep disorders [69,70], upcoming studies should also include measurements of these psychological variables and, thus, control their potential mediating/moderating role.

## 6. Conclusions

Our study further highlights the potential underlying mechanisms through which body composition, especially BMI, has a significant impact on objective and subjective sleep. According to our findings, sedentary behavior is the link/risk factor mediating the adverse consequences of high BMI on short sleep duration in healthy young men. Other potential modifiable risk factors such as physical activity and diet, although not mediating the impact of BMI on sleep, are also independently related to both these current public health concerns, i.e., obesity and sleep curtailment. Due to the vast and adverse health-related consequences of these two conditions and, thus, the resulting resource utilization and/or healthcare costs, the identification of these potential modifiable risk factors and affected young patients should become an essential goal for researchers and clinicians. To this end, researchers and healthcare professionals from different fields should collaborate in multidisciplinary research and interventions. Not until the complex association between body composition and sleep in young population is properly understood will it be possible to establish appropriate therapeutic goals addressing and limiting the early morbidity and mortality that the interactions of health conditions such as obesity and short sleep duration certainly determine.

## Figures and Tables

**Table 1:** Descriptive characteristic of participants.

	*n*	All			*n*	Men			*n*	Women		
Age (years)	187	22.07	±	2.19	66	22.25	±	2.24	121	21.97	±	2.17
Sleep quality												
Total sleep time (min)	187	385.63	±	44.02	66	378.38	±	44.70	121	389.58	±	43.32
Sleep efficiency (%)	187	86.99	±	5.01	66	85.86	±	5.17	121	87.61	±	4.83 *
Wake after sleep onset (min)	187	59.68	±	26.29	66	65.53	±	31.81	121	56.49	±	22.24 *
PSQI total score	179	5.63	±	2.60	61	6.02	±	2.66	118	5.42	±	2.55
Anthropometry and body composition												
Body mass index (kg/m^2^)	178	24.97	±	4.80	58	26.84	±	5.26	120	24.06	±	4.29 *
Waist–hip ratio	147	0.80	±	0.10	48	0.87	±	0.09	87	0.76	±	0.08 *
Waist–height ratio	170	0.48		0.08	55	0.51		0.08	115	0.47		0.07 *
Lean mass (kg)	178	41.93	±	9.81	58	53.11	±	7.37	120	36.53	±	5.19 *
Lean mass index (kg/m^2^)	178	14.74	±	2.44	58	17.14	±	2.09	120	13.58	±	1.62 *
Fat mass (kg)	178	24.83	±	8.89	58	25.37	±	10.78	120	24.57	±	7.85
Fat mass (%)	178	35.50	±	7.37	58	30.13	±	7.30	120	38.09	±	5.87 *
Fat mass index (kg/m^2^)	178	8.83	±	3.08	58	8.19	±	3.43	120	9.14	±	2.86
Visceral adipose tissue mass (g)	150	340.95	±	179.43	49	427.10	±	179.18	101	299.15	±	164.80 *
Sedentariness and physical activity levels
Sedentary time (min)	187	926.9	±	55.1	66	935.1	±	59.9	121	922.4	±	51.9
Light physical activity (min)	187	24.5	±	12.7	66	24.7	±	12.6	121	24.4	±	12.7
Moderate physical activity (min)	187	59.8	±	24.7	66	56.5	±	23.2	121	61.6	±	25.4
Vigorous physical activity (min)	187	1.8	±	3.3	66	1.6	±	2.1	121	1.9	±	3.8
Moderate–vigorous physical activity (min)	187	61.7	±	26.5	66	58.2	±	23.8	121	63.6	±	27.8
Dietary intake												
Energy intake (kcal)	170	1867.0	±	532.7	59	2104.3	±	519.1	111	1741.0	±	497.7 *
Fat intake (g)	170	83.4	±	29.2	59	94.1	±	29.2	111	77.7	±	27.7 *
Protein intake (g)	170	75.6	±	24.0	59	91.1	±	26.1	111	67.3	±	18.2 *
Carbohydrates intake (g)	170	197.9	±	66.8	59	214.1	±	71.0	111	189.3	±	63.1 *

Data are shown as means ± standard deviation. * Significant differences between sexes obtained from a Student’s unpaired-samples *t*-test (*p* < 0.05). PSQI, Pittsburgh sleep quality index.

**Table 2:** Unstandardized regression coefficients (standard errors) with confidence intervals of BMI estimating sedentariness and total sleep time including sex as a moderator variable.

	Sedentariness (*M*)			Total Sleep Time (*Y*)		
		Coefficient (SE)	95% CI		Coefficient (SE)	95% CI
**Body mass index (*X*)**	a_1_	83.8837 ** (28.1842)	28.2498, 139.5175	c’	−8.8941 * (4.3049)	−17.3914, −0.3969
**Sex (*V*)**	a_2_	51.4133 * (24.9181)	2.2266, 100.6000			
X*W	a_3_	−46.0177 ** (16.1402)	−77.8774, −14.1581			
**Sedentariness (*M*)**				b	−0.5511 *** (0.0430)	−0.6360, −0.4663
**Moderated Mediation Index**					25.3618 (0.0013)	7.8916, 43.8275

* *p* < 0.05, ** *p* < 0.01, *** *p* < 0.001. CI, confidence interval.

## Supplementary Materials

The following are available online at https://www.mdpi.com/2077-0383/9/5/1560/s1: Table S1. Association of body mass index, waist–hip ratio, lean mass index, fat mass index, and visceral adipose tissue mass with total sleep time, sleep efficiency, wake after sleep onset, and Pittsburgh total score before and after adjusting for sex (Model 0 and Model 1, respectively); Table S2. Correlations of sleep parameters with sedentary time, physical activity levels, and dietary intake; Table S3. Correlations of body composition with sedentary time, physical activity variables, and dietary intake; Table S4. Correlations of sedentary time, physical activity, and dietary intake; Figure S1. Mediation model of the relationship of BMI and total sleep time (min) with energy intake (**A**), fat intake (**B**), protein intake (**C**), carbohydrates intake (**D**), sedentary time (**E**), and moderate–vigorous physical activity (**F**) as mediator variables.
