# Supplementary material for: Body Composition Impact on Sleep in Young Adults: The Mediating Role of Sedentariness, Physical Activity, and Diet
Source: J Clin Med. 2020 May 21;9(5):1560. doi: 10.3390/jcm9051560 (PMC7290677; doi:10.3390/jcm9051560)
Supplement: Supplementary file 1 [file jcm-09-01560-s001.pdf]

Supplementary Materials

Table S1. Association of body mass index (kg/m2), waist-hip ratio, waist-height ratio lean mass index (kg/m2), fat mass index (kg/m2) and visceral adipose tissue mass (g) with the total sleep time, sleep efficiency, wake after sleep onset and Pittsburgh total score before and after adjusting for sex (Model 0 and Model 1, respectively). P from multiple regression analyses.

	Total sleep time					
	Model 0			Model 1		
	β	R^2	P	β	R^2	P
Body mass index (kg/m^2)	-0.165	0.027	**0.029**	-0.124	0.093	0.092
Waist-hip ratio	-0.222	0.049	**0.007**	-0.203	0.050	**0.039**
Waist-height ratio	-0.170	0.029	**0.028**	-0.134	0.041	0.096
Lean mass index (kg/m^2)	-0.219	0.048	**0.004**	-0.115	0.489	**0.039**
Fat mass index (kg/m^2)	-0.071	0.005	0.353	-0.094	0.028	0.216
Visceral Adipose Tissue (g)	-0.149	0.022	0.070	-0.102	0.127	0.196
	Sleep efficiency					
	Model 0			Model 1		
	β	R^2	P	β	R^2	P
Body mass index (kg/m2)	-0.086	0.007	0.259	-0.036	0.079	0.631
Waist-hip ratio	-0.174	0.030	**0.037**	-0.081	0.050	0.409
Waist-height ratio	-0.068	0.005	0.380	-0.013	0.036	0.876
Lean mass index (kg/m^2)	-0.188	0.035	**0.013**	-0.064	0.48	0.255
Fat mass index (kg/m^2)	0.012	0.001	0.872	-0.014	0.020	0.858
Visceral Adipose Tissue (g)	-0.061	0.004	0.462	0.013	0.117	0.868
	Wake after sleep onset					
	Model 0			Model 1		
	β	R^2	P	β	R^2	P
Body mass index (kg/m2)	0.024	0.001	0.754	-0.028	0.079	0.710
Waist-hip ratio	0.169	0.029	**0.042**	0.069	0.052	0.481
Waist-height ratio	-0.007	0.001	0.925	-0.71	0.040	0.380
Lean mass index (kg/m^2)	0.144	0.021	0.058	0.019	0.476	0.733
Fat mass index (kg/m^2)	-0.068	0.005	0.370	-0.044	0.021	0.564
Visceral Adipose Tissue (g)	0.013	0.001	0.878	-0.067	0.121	0.405
	Pittsburg total score					
	Model 0			Model 1		
	β	R^2	P	β	R^2	P
Body mass index (kg/m2)	0.088	0.008	0.253	0.045	0.099	0.543
Waist-hip ratio	0.063	0.004	0.457	0.010	0.011	0.918
Waist-height ratio	0.067	0.005	0.393	0.025	0.023	0.760
Lean mass index (kg/m^2)	0.145	0.021	0.058	0.044	0.532	0.407
Fat mass index (kg/m^2)	0.014	0.001	0.859	0.033	0.019	0.671
Visceral Adipose Tissue (g)	0.125	0.016	0.136	0.088	0.125	0.270

Table S2. Correlations of sleep parameters with sedentary time, physical activity levels and dietary intake.

	Total sleep duration (min)			Sleep efficiency (%)			Wake after sleep onset (min)			PSQI total score		
	All	Men	Women	All	Men	Women	All	Men	Women	All	Men	Women
Sedentary time and physical activity												
Sedentary time (min)	**-0.693****	**-0.716****	**-0.673****	-0.064	0.131	-0.166	-0.109	-0.285*	0.007	0.032	-0.002	0.006
Light physical activity (min)	-0.031	-0.049	-0.019	0.024	-0.026	0.056	-0.058	-0.024	-0.090	0.019	-0.031	-0.063
Moderate physical activity (min)	-0.002	0.013	-0.029	0.143	-0.009	**0.203***	**-0.156***	0.004	**-0.251****	0.035	0.107	-0.007
Vigorous physical activity (min)	0.033	-0.007	0.041	0.127	-0.020	0.175	-0.127	0.004	**-0.194***	-0.071	-0.200	-0.040
Moderate-vigorous physical activity (min)	0.002	0.012	-0.021	**0.149***	-0.011	**0.209***	**-0.161***	0.004	**-0.256****	0.030	0.086	-0.011
Dietary intake												
Energy intake (kcal)	0.043	0.094	0.071	0.018	0.170	0.020	-0.046	-0.185	-0.050	0.055	-0.132	0.107
Fat intake (g)	0.074	0.150	0.079	0.044	0.179	0.042	-0.059	-0.182	-0.056	-0.029	-0.140	0.123
Protein intake (g)	0.024	0.074	0.090	-0.036	0.094	0.032	0.031	-0.107	-0.028	-0.018	-0.136	0.036
Carbohydrates intake (g)	0.024	0.039	0.041	0.030	0.136	0.005	-0.069	-0.157	-0.047	0.034	-0.070	0.079

PSQI, Pittsburgh sleep quality index.
* $p < 0.05$, ** $p < 0.01$

Table S3. Correlations of body composition with sedentary time, physical activity variables, and dietary intake.

	Sedentary time (min)	LPA (min)	MPA (min)	VPA (min)	MVPA (min)	Energy intake (kcal)	Fat intake (g)	Protein intake (g)	Carbohydrates intake (g)
Body mass index (kg/m^2)									
All	0.137	0.065	-0.021	0.008	-0.042	-0.042	-0.083	0.040	-0.027
Men	**0.271***	-0.076	-0.231	-0.163	-0.240	**-0.319***	**-0.341***	**-0.299***	-0.163
Women	-0.010	0.158	0.136	0.086	0.136	0.000	-0.013	0.037	-0.006
Waist-Hip ratio									
All	**0.234****	-0.011	-0.156	-0.126	-0.162	0.060	0.041	**0.203***	-0.023
Men	**0.355***	-0.179	-0.258	-0.207	-0.268	-0.250	-0.179	-0.193	-0.268
Women	0.087	0.094	-0.026	-0.119	-0.037	-0.017	-0.020	0.027	-0.021
Waist-Height ratio									
All	**0.178***	0.061	-0.071	-0.033	-0.070	-0.045	-0.070	0.063	-0.045
Men	**0.311***	-0.091	-0.264	-0.180	**-0.273***	-0.254	-0.228	-0.238	-0.165
Women	0.023	0.170	0.083	0.035	0.081	-0.044	-0.068	0.051	-0.033
Lean mass (kg)									
All	0.111	0.039	0.004	0.035	0.008	**0.266****	0.207*	**0.414****	0.147
Men	0.040	0.080	-0.024	0.063	-0.018	-0.147	-0.152	-0.073	-0.107
Women	-0.033	0.115	**0.225***	0.168	**0.229***	**0.265****	**0.260****	**0.204***	0.191
Lean mass index (kg/m^2)									
All	0.127	0.069	0.008	0.052	0.014	**0.174***	0.101	**0.316****	0.098
Men	0.100	0.076	-0.084	0.037	-0.079	-0.207	-0.265	-0.239	-0.088
Women	0.003	0.146	**0.203***	0.163	**0.208***	0.099	0.087	0.143	0.060
Fat mass (kg)									
All	0.127	0.009	-0.057	-0.058	-0.060	-0.109	-0.115	-0.072	-0.067
Men	**0.299***	-0.158	-0.257	-0.247	**-0.272***	**-0.324***	**-0.315***	-0.239	-0.185
Women	-0.013	0.112	0.074	0.008	0.068	0.029	0.025	0.018	0.017
Fat mass (%)									
All	0.065	-0.006	-0.057	-0.089	-0.065	**-0.308****	**-0.266****	**-0.357****	**-0.192***
Men	**0.379****	-0.195	**-0.281***	**-0.306***	**-0.301***	**-0.349***	**-0.329***	-0.261	-0.206
Women	0.005	0.072	-0.029	-0.088	-0.039	-0.104	-0.096	-0.070	-0.093
Fat mass index (kg/m^2)									
All	0.107	0.026	-0.048	-0.046	-0.051	**-0.204***	**-0.204***	**-0.196***	-0.122
Men	**0.327***	-0.167	**-0.284***	-0.254	**-0.299***	**-0.350***	**-0.349***	**-0.303***	-0.188
Women	-0.004	0.123	0.059	0.005	0.055	-0.051	-0.060	-0.022	-0.042
Visceral Adipose Tissue (g)									
All	**0.187***	0.114	-0.125	-0.128	-0.132	-0.022	-0.061	0.065	-0.011
Men	**0.328***	-0.056	-0.214	**-0.290***	-0.232	**-0.284***	-0.271	-0.218	-0.168
Women	0.047	**0.227***	-0.034	-0.071	-0.040	-0.031	-0.062	-0.038	0.003

LPA, light physical activity; MPA, moderate physical activity, VPA, vigorous physical activity; MVPA, moderate-vigorous physical activity.
* $p < 0.05$, ** $p < 0.01$

Table S4. Correlations for sedentary time, physical activity and dietary intake.

	Sedentary time (min)	LPA (min)	MPA (min)	VPA (min)	MVPA (min)
Sedentary time					
All		**-0.395****	**-0.531****	**-0.340****	**-0.538****
Men		**-0.392****	**-0.517****	-0.195	**-0.521****
Women		**-0.404****	**-0.536****	**-0.411****	**-0.547****
Energy intake (kcal)					
All	-0.112	0.015	0.126	0.039	0.124
Men	-0.142	0.160	0.062	-0.096	0.051
Women	-0.143	-0.108	**0.222***	0.099	**0.222***
Fat intake (g)					
All	-0.101	-0.039	0.094	-0.004	0.090
Men	-0.099	0.121	0.050	-0.092	0.039
Women	-0.146	-0.178	0.170	0.032	0.166
Protein intake (g)					
All	-0.082	0.060	0.128	0.083	0.131
Men	-0.095	0.118	0.189	0.014	0.184
Women	-0.075	-0.044	0.176	0.115	0.180
Carbohydrates intake (g)					
All	-0.080	0.020	0.083	0.047	0.084
Men	-0.117	0.092	-0.034	-0.089	-0.041
Women	-0.168	-0.019	**0.214***	0.124	**0.217***

LPA, light physical activity; MPA, moderate physical activity, VPA, vigorous physical activity; MVPA, moderate-vigorous.

* $p < 0.05$, ** $p < 0.01$

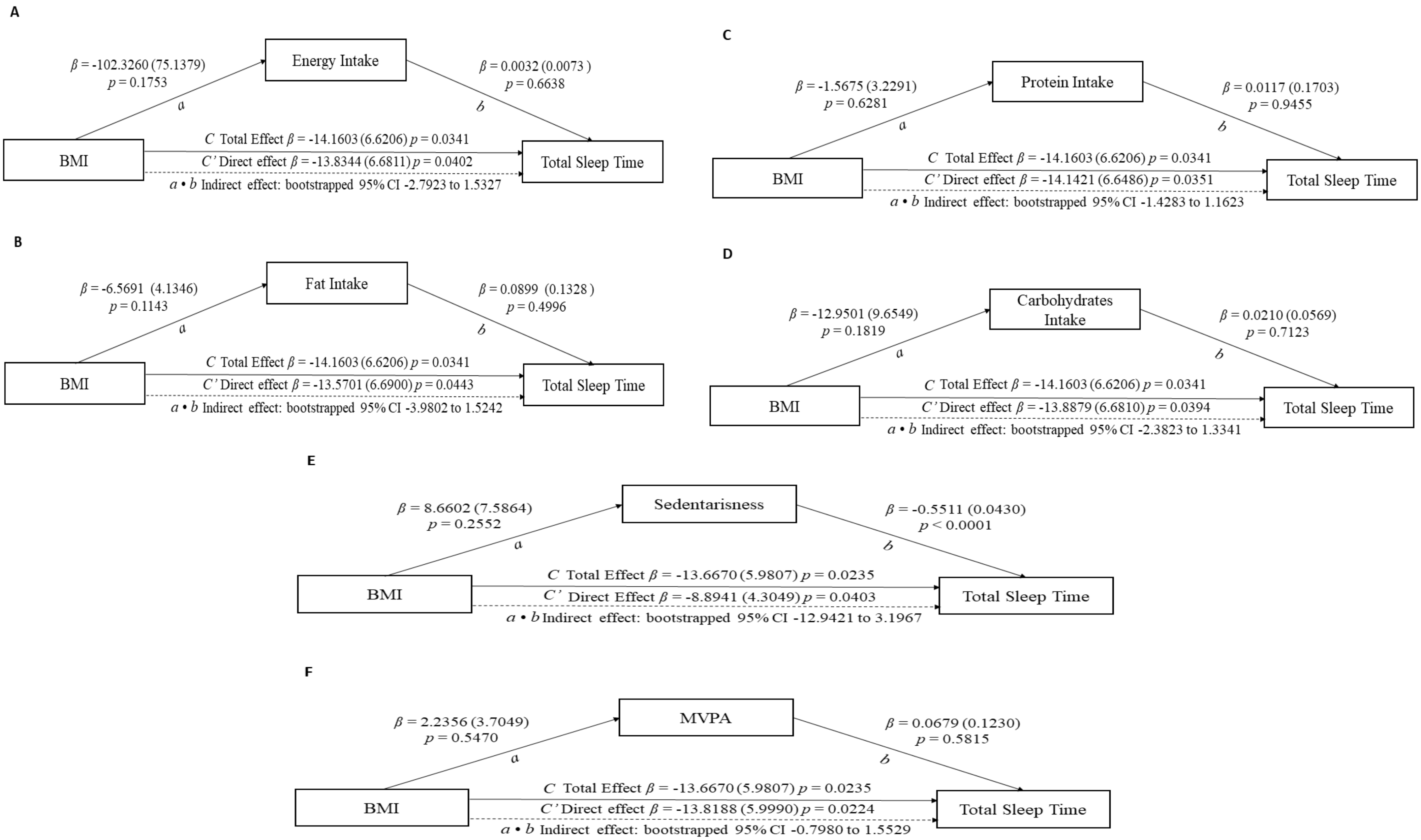

Figure S1. Mediation model of the relationship between BMI and Total Sleep Time (min) with energy intake (A), fat intake (B), protein intake (C), carbohydrates intake (D), sedentary time (E), and moderate-vigorous physical activity (F) as mediator variables. Paths *a*, *b*, *c*, and *c'* are presented as unstandardized coefficients (standard error). [Lower-limit CI; upper-limit CI], lower and upper levels for 95% bias-corrected CIs of the indirect effects based on 5,000 bootstraps. BMI, body mass index; MVPA, moderate-vigorous physical activity; CI, confidence interval.
